# Perioperative utilization of JAK inhibitors in Perianal Fistulizing Crohn's disease

**DOI:** 10.1002/jpr3.70101

**Published:** 2025-10-28

**Authors:** Alexander Lyons, Lindsey Lawrence, Samantha Saul

**Affiliations:** ^1^ Department of Pediatric Gastroenterology University of Michigan Ann Arbor Michigan USA

**Keywords:** inflammatory bowel disease, ostomy takedown, upadacitinib

## Abstract

With the recent approval of small molecule drugs such as upadacitinib in adult inflammatory bowel disease (IBD), their utilization is becoming more common; however, there is limited data on perioperative risks or optimal timing of cessation and resumption to mitigate flares. Current recommendations suggest holding these medications for 14 days postoperatively for IBD‐related surgeries. We present a 17‐year‐old female with severe fistulizing perianal and rectosigmoid Crohn's disease who required diverting sigmoidostomy for her perianal disease. Her disease was controlled on upadacitinib for 1 year; however, she had reoccurrence of fistulizing disease with perianal abscess after stopping upadacitinib for 14 days following sigmoidostomy takedown. This 14‐day timeline puts patients at risk of resumption of active disease, with fistula recurrence being a disease subset that may not be able to be salvaged with medication resumption alone. Shared decision‐making is crucial before deciding how long to withhold these medications, but more research is needed to provide concrete guidelines.

## 1 INTRODUCTION

Surgical management of immunosuppressed inflammatory bowel disease (IBD) patients requires thoughtful consideration to minimize infectious risks and the risks of disease recurrence. Medications in the tumor necrosis factor inhibitor (TNFi) category have been the best studied with the multicenter prospective cohort PUCCINI study, showing that neither patient‐reported use of anti‐TNF agents within 12 weeks before surgery nor detectable serum drug concentrations were independent risk factors for any postoperative infection or surgical site infection. Perioperative risks were best mitigated by focusing on other elements, such as minimizing corticosteroids and smoking, along with controlling diabetes.^1^ With the more recent approval of small molecule drugs such as upadacitinib in adult IBD, its off‐label use in pediatrics has increased in patients with refractory IBD; however, pediatric studies involving JAK inhibitors (JAKi) for IBD are still limited. Within adult literature, there is limited data on perioperative risks or optimal timing of cessation and resumption to mitigate these risks in IBD patients. Current recommendations suggest holding these medications for 14 days postoperatively for IBD‐related surgeries.^2^ This is a very important consideration given the short half‐life of 8–14 h for this medication.

## 2 CASE REPORT

A 17‐year‐old female with past medical history of posttraumatic stress disorder and anxiety was initially diagnosed with severe fistulizing perianal and rectosigmoid Crohn's disease in June 2020 (age 12 at time of diagnosis; Paris Classification: A1b L2 B3 P G0). She had a diverting sigmoidostomy in March 2021 as her perianal disease persisted despite well‐dosed infliximab 10 mg/kg every 4 weeks with levels >10 mcg/mL for over 6 months. She subsequently had ongoing perianal disease with recurrent abscesses despite simultaneous use of ustekinumab 90 mg every 4 weeks started in April 2021, adalimumab 80 mg every 2 weeks started in July 2021, and 8‐week course of hyperbaric oxygen treatments from October 2021 to December 2021.

She only began to see improvement in her disease when she was induced with upadacitinib 30 mg daily along with ustekinumab 90 mg every 4 weeks in June 2022. At that time, upadacitinib was started at 30 mg daily based on an expert second opinion recommendation as the patient was on ustekinumab at the time. Adalimumab was discontinued to avoid further immunosuppression due to previous failure with TNFi. With ongoing clinical and biochemical remission with upadacitinib and ustekinumab, it was thought that upadacitinib was the reason for the remission, as the patient had previously failed with simultaneous use of ustekinumab with adalimumab.

In December 2023, she was subsequently transitioned to upadacitinib 30 mg monotherapy with continued clinical and biochemical remission as well as monitoring with magnetic resonance imaging of the pelvis (MR Pelvis) to ensure perianal disease was stable. MR Pelvis from June 2024 showed a small residual left intersphincteric fistulous tract ranging between the 3:00 and 5:00 positions without abscesses. Subsequent esophagogastroduodenoscopy, ileoscopy, and flexible sigmoidoscopy in August 2024 showed mucosal healing with what seemed like a remnant of a prior fistula posterior to the rectum. As she was complaining of possible fistula drainage following endoscopy, she underwent exam under anesthesia with surgery in November 2024 for fistula interrogation and was found instead to have pilonidal disease without any signs of perianal Crohn's disease. She subsequently underwent a Gips (pit picking) procedure. In December 2024, the magnetic resonance enterography and pelvis were without concern for fistula or abscess.

**Figure 1:**
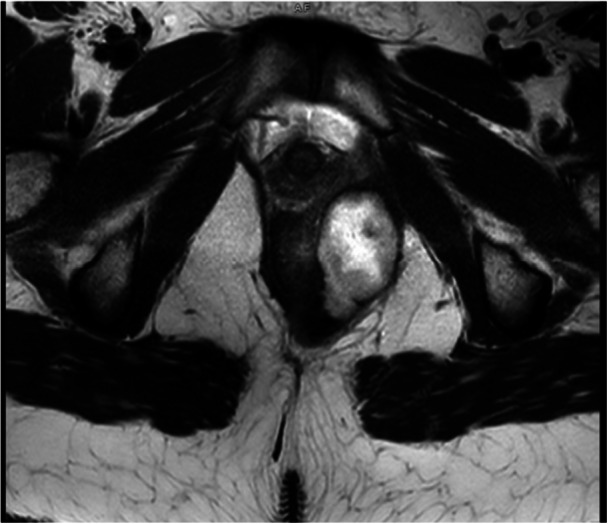
5.5 × 3.3 × 4.1 cm (antero‐posterior, transverse, and cranio‐caudal) measuring abscess with a necrotic center and marked diffusion restriction in the left perianal region extending posteriorly to the anus without evidence of a definite fistulous tract.

At that time, it was felt that her IBD was well‐controlled for just over a year, and she subsequently underwent sigmoidostomy take‐down in February 2025. She was recommended to hold upadacitinib for 14 days postoperatively. Twenty‐two days after her procedure, she started to complain of rectal pain. Twenty‐eight days postprocedure, her symptoms progressed to include foul‐smelling, mucoid green rectal drainage after her bowel movements. She was afebrile, had no hematochezia or abdominal pain. At presentation, 29 days after her procedure, her C‐reactive protein and erythrocyte sedimentation rate were elevated at 6.1 mg/dL (reference range: <0.6 mg/dL) and 98 mm/h (reference range: 0–20 mm), respectively. MRE showed a 5.5 × 3.3 × 4.1 cm abscess in the left perianal region (Figure 1).

**Figure 2:**
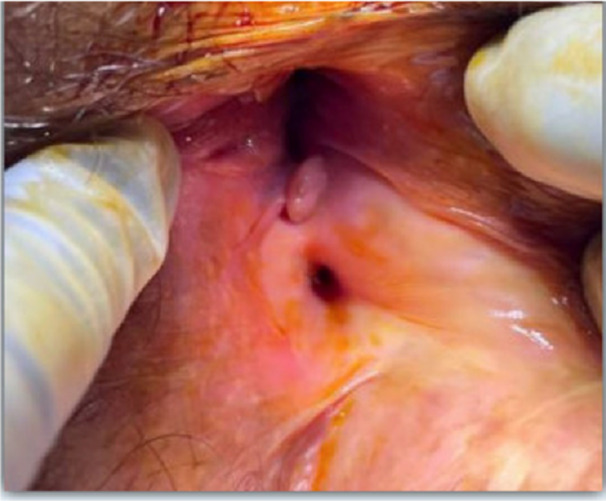
Left posterior perianal/perirectal abscess (5 o'clock in supine). Fistula‐in‐ano at 5 o'clock that is deep (posterior to sphincter) with a communication to a perirectal abscess in line from an opening above the dentate line (covered by mucosal tag) with another opening deeper in the rectum.

She underwent incision and drainage and had a seton placed through a fistula in the 5–6 o'clock position (Figure 2). This was felt to be a new fistula that developed postoperative in the setting of upadacinitib withdrawal, as it was not seen on prior imaging or endoscopy. Upadacitinib was then resumed at 45 mg for reinduction.

## 3 DISCUSSION

There is limited data regarding the use of JAKi in perioperative IBD patients. The FDA black box warning in 2021 included all JAK inhibitors, describing the risk of serious myocardial events, cancer, venous thromboembolic events (VTEs), and death. Current safety data on JAKi in perioperative IBD patients have focused on adult patients with ulcerative colitis exposed to tofacitinib undergoing total abdominal colectomy. The study found the rate of VTE rate was 13% however studies on upadacitinib, like our case, are limited and theoretically may be lower due to selective binding of JAK receptors.^2^ In fact, large trials have shown that upadacitinib 15 mg once daily in rheumatoid arthritis patients had a similar safety profile to adalimumab except in areas for Herpes Zoster infection.^3^

Data for perioperative medication timing in IBD patients is even more limited. Preoperative recommendations in rheumatological patients undergoing total hip arthroplasty or total knee arthroplasty currently recommends holding JAKi for at least 3 days before surgery.^4^ While withholding these medications for 3 days preoperatively is reasonable based on its short half‐life of 8–14 h, little is known about the duration of immunosuppression after the drug is withheld. Current recommendations in IBD regarding holding medications for 14 days postoperatively are largely based on rheumatological adult studies undergoing orthopedic procedures.^5^, ^6^, ^7^ This timeline puts patients at risk of resumption of active disease, with fistula recurrence being a disease subset that may not be able to be salvaged with medication resumption alone.

Recent rheumatological data points out the risk of disease flare during prolonged discontinuation. In one prospective study the median preoperative and postoperative JAKi withdrawal periods were 1 day and 4.5 days respectively with 23.3% of patients flaring.^8^ The preoperative withdrawal period was not associated with flare in patients who had stopped their JAKi for ≥4 days compared to ≤1 day. In contrast, the postoperative withdrawal period of JAKi was significantly associated with flares if JAKi was held >14 days compared to ≤7 days.^9^ These studies show how shortening the postoperative JAKi withdrawal period may be essential to prevent flares. It is also important to note the differences in applying this recommendation to IBD patients as orthopedic procedures require exquisitely sterile environments compared to intestinal surgery.

## 4 CONCLUSION

In summary, our patient had reoccurrence of perianal disease following ostomy takedown associated with holding upadacinitib. Reoccurrence is more likely if there was active perianal disease at the time of the surgery, especially fistulas that have not been adequately healed or managed; however, the rapid onset of fistulizing disease following sigmoidostomy takedown is unusual, with reoccurrence typically occurring 2–6 months following restoration of bowel continuity.^10^ Our patient had an endoscopic evaluation 6 months before takedown, exam under anesthesia with surgery 3 months before takedown, and MRE 2 months before takedown without concern for active or fistulizing disease, raising concern that the length of holding upadacinitib contributed to her disease flare. Closer scope evaluation to ostomy takedown would help clarify if there was any active disease or if fistula reoccurrence was related to medication discontinuation. For surgeries involving IBD patients, shared decision‐making is crucial before deciding how long to withhold JAKi, but more research is needed to provide concrete recommendations.

## CONFLICT OF INTEREST STATEMENT

The authors declare no conflicts of interest.

## ETHICS STATEMENT

The authors thank the patient and her family for their informed consent to publish this case.

## References

[1] Cohen BL , Fleshner P , Kane SV , et al. Prospective cohort study to investigate the safety of preoperative tumor necrosis factor inhibitor exposure in patients with inflammatory bowel disease undergoing intra‐abdominal surgery. Gastroenterology. 2022;163(1):204‐221. 35413359 10.1053/j.gastro.2022.03.057

[2] Lightner AL , Vaidya P , Holubar S , et al. S0732 Perioperative safety of tofacitinib in surgical inflammatory bowel disease patients. Am J Gastroenterol. 2020;115:S367‐S368.

[3] Blockmans D , Penn SK , Setty AR , et al. A phase 3 trial of upadacitinib for giant‐cell arteritis. N Engl J Med. 2025;392(20):2013‐2024. 40174237 10.1056/NEJMoa2413449

[4] Goodman SM , Springer BD , Chen AF , et al. 2022 American College of Rheumatology/American Association of Hip and Knee Surgeons guideline for the perioperative management of antirheumatic medication in patients with rheumatic diseases undergoing elective total hip or total knee arthroplasty. Arthritis Care Res. 2022;74(9):1399‐1408. 10.1002/acr.24893 35718887

[5] Cohen BL , Lincango E , Holubar SD . How to manage targeted immune suppressants (biologics and oral small‐molecule drugs) perioperatively for inflammatory bowel disease and non‐inflammatory bowel disease surgery. Clin Gastroenterol Hepatol. 2023;21(5):1148‐1151.e1. 36934049 10.1016/j.cgh.2023.01.042

[6] James WA , Rosenberg AL , Wu JJ , et al. Full guidelines‐from the Medical Board of the National Psoriasis Foundation: perioperative management of systemic immunomodulatory agents in patients with psoriasis and psoriatic arthritis. J Am Acad Dermatol. 2024;91(2):251.e1‐251.e11. 10.1016/j.jaad.2024.03.008 38499181

[7] Russell LA , Craig C , Flores EK , et al. Preoperative management of medications for rheumatologic and HIV diseases: Society for Perioperative Assessment and Quality Improvement (SPAQI) consensus statement. Mayo Clin Proc. 2022;97(8):1551‐1571. 35933139 10.1016/j.mayocp.2022.05.002

[8] Yoshida T , Onishi A , Hasegawa Y , et al. POS0850 The effect of perioperative Janus kinase inhibitor withdrawal on surgical site infection and flare of rheumatoid arthritis in orthopedic surgery: a multi‐center observational study. Ann Rheum Dis. 2023;82:726‐727.

[9] Nishida K , Harada R , Nasu Y , et al. Influence of Janus kinase inhibitors on early postoperative complications in patients with rheumatoid arthritis undergoing orthopaedic surgeries. Mod Rheumatol. 2024;34(3):466‐473. 37279573 10.1093/mr/road047

[10] Singh S , Ding NS , Mathis KL , et al. Systematic review with meta‐analysis: faecal diversion for management of perianal Crohn's disease. Aliment Pharmacol Ther. 2015;42(7):783‐792. 26264359 10.1111/apt.13356 PMC6698449

